# Evaluation of biological potential of selected species of family Poaceae from Bahawalpur, Pakistan

**DOI:** 10.1186/s12906-018-2092-1

**Published:** 2018-01-24

**Authors:** Iram Fatima, Sobia Kanwal, Tariq Mahmood

**Affiliations:** 1 Department of Plant Sciences, Faculty of Biological Sciences, Quaid-i-Azam University, Islamabad, 45320 Pakistan; 2 Department of Zoology, University of Gujrat Sub-Campus Rawalpindi, Gujrat, Pakistan

**Keywords:** Fodder grasses, Phytochemical analysis, Antioxidant activity, p1391Z plasmid DNA, Antibacterial activity, Antifungal activity, Brine shrimps’ Cytotoxicity

## Abstract

**Background:**

Oxidative stress as well as bacterial and fungal infections are common source of diseases while plants are source of medication for curative or protective purposes. Hence, aim of study was to compare the pharmacological potential of seven grass species in two different solvents i.e. ethanol and acetone.

**Methods:**

Preliminary phytochemical tests were done and antioxidant activities were evaluated using ELISA and their IC50 values and AAI (%) were recorded. ANOVA was used for statistical analyses. DNA damage protection assay was done using p1391Z plasmid DNA and DNA bands were analyzed. Antimicrobial activity was done via disc diffusion method and MIC and Activity Index were determined. Cytotoxic activity was carried out using the brine shrimps’ assay and LC50 values were calculated using probit analysis program.

**Results:**

Phytochemical studies confirmed the presence of secondary metabolites in most of the plant extracts. Maximum antioxidant potential was revealed in DiAEE, DiAAE (AAI- 54.54% and 43.24%) and DaAEE and DaAAE (AAI- 49.13% and 44.52%). However, PoAEE and PoAAE showed minimum antioxidant potential (AAI- 41.04% and 34.11%). SaSEE, DiAEE and ElIEE showed very little DNA damage protection activity. In antimicrobial assay, DaAEE significantly inhibited the growth of most of the microbial pathogens (nine microbes out of eleven tested microbes) among ethanol extracts while DaAAE and ImCAE showed maximum inhibition (eight microbes out of eleven tested microbes) among acetone plant extracts. However, PoAEE and PoAAE showed least antimicrobial activity. *F. oxysporum* and *A. niger* were revealed as the most resistant micro-organisms. ImCEA and ImCAE showed maximum cytotoxic potential (LC50 11.004 ppm and 7.932 ppm) as compared to the other plant extracts.

**Conclusion:**

Fodder grasses also contains a substantial phenols and flavonoids contents along with other secondary metabolites and, hence, possess a significant medicinal value. Ethanol extracts showed more therapeutic potential as compared to the acetone extracts. This study provides experimental evidence that the selected species contains such valuable natural compounds which can be used as medicinal drugs in future.

## Background

Herbal drugs have got a distinct place since ancient times. Both human and plant use history are of a similar age yet the proof concerning the use of plants is found in Ayurveda between 2500 and 600 BC [1]. The diverse medicinal plants are utilized for food and treatment of various ailments from their discovery. Certainly, Pakistan is a well-develop ecological zone, in which medicinal plants are utilized as a medicine for human as well as animals. Bahawalpur region is in one of the nine divisions of the Punjab province and its total area is 18,000 sq. miles. Bahawalpur was constructed on the southern bank of Sutlej River [2]. Poaceae or the grass family is the fifth largest family of flowering plants containing about 50 tribes, 660 genera and 10,000 species [3]. In Pakistan, Poaceae is represented by 158 genera and 492 species [4] and is of incredible economic and medicinal importance. Some plants of family Poaceae are used as a medication for hypertension, antidiabetic, anti-inflammatory, anthelmintic, antiulcer, diuretic and antioxidant [5].

*Dichanthium annulatum* (Forsk.) Stapf, generally known as ‘marvel grass’ is classified within the Order of Poales and is used as forage due to its simple and cheaper development from seed [6]. The grass is also used for dysentery and menorrhagia [7]. *Eleusine indica* L. is commonly known as ‘wire grass or goose grass’ and is used in traditional medicine as a diuretic, anti-helminthic, anti-cancer, febrifuge, hypertension, kidney problems and for treating influenza and cough [8, 9]. Similarly, *Poa annua* L. (annual bluegrass) is a cosmopolitan weed with a high genotypic and phenotypic variability. *Dactyloctenium aegyptium* L. has anti-oxidant, anti-inflammatory, anticancer, antipyretic properties and antimicrobial activity and is used for treating small pox, wounds and ulcers [10].

*Saccharum spontaneum* L. generally known as ‘wild sugarcane’ is used to treat diseases such as vomiting, anaemia, abdominal disorders and obesity. The roots are used as astringent, emollient, diuretic, tonic and for treating dyspepsia, burning sensation, piles, respiratory troubles etc. [11]. Leaves are employed for cathartic and diuretics. However, the whole plant possesses antidiarrheal, CNS depressant and antiurolithiatic activity [12, 13]. *Vetiveria zizanioides* (L.) is usually known as ‘Khas-Khas’ and has thick adventitious roots which are stimulant, stomachic and used in fevers and inflammations [14]. Vetiver grass is also grown for the production of oil which is used in perfumery and aromatherapy [15]. *Imperata cylindrica* L., commonly known as thatch grass or cogon grass, possess immunomodulatory, neuroprotective, anticancer, and antioxidant activities [16, 17].

To date, a few studies of the selected grass species relevant to their antifungal, antioxidant and brine shrimps’ cytotoxic activity have been reported. Natural products require strong evaluation of their pharmacological abilities. Accordingly, this study was conducted to assess the biological potential and DNA damage protection assay of the selected grasses collected from different areas of Bahawalpur to evaluate their pharmacological potential.

## Methods

### Collection and preparation of plant extracts

*D. annulatum* (Acc. No. 129851)*, E. indica* (Acc. No. 129854)*, P. annua* (Acc. No. 129850)*, D. aegyptium* (Acc. No. 129846)*, S. spontaneum* (Acc. No. 129841)*, V. zizanioides* (Acc. No. 129844) and *I. cylindrica* (Acc. No. 129842) were collected from Lal Suhanra National Park and Islamia University of Bahawalpur, Pakistan during the month of March, 2016 and accession numbers were allotted by Herbarium of Pakistan, Quaid-e-Azam University, Islamabad. Aerial parts of plants were selected because of their ethno-medicinal importance. Plants were washed, air-dried, powdered and extracted with ethanol and acetone (50 g/500 mL each) for two days and then filtered and evaporated using a rotary evaporator (BUCHI Rotavapor R-220) and then stored at 4 °C for further use [18].

### Preliminary phytochemical tests

Various qualitative tests are performed for establishing profile of the plant extracts for its chemical composition. So, preliminary phytochemical tests of the plant extracts were done to confirm the presence of secondary metabolites, using standard procedures for the detection of alkaloids (Mayer’s reagent), cardiac glycosides (Salkowski test), saponins (Foam test), flavonoids (Alkaline reagent test), phenols (Ferric chloride test), tannins (Gelatin test), steroids and terpenoids (Libermann’s test), anthocyanins (2 ml extract + 2 ml HCl + NH_3_ – pinkish red to bluish violet colour) and coumarins (2 ml extract + 3 ml NaOH – yellow colour) [18, 19].

### Total phenols and flavonoids contents

The content of total phenols was determined using Folin-Ciocalteu reagent [20]. Calibration curve was made by mixing gallic acid with 90 μl of Folin-Ciocalteu reagent and 90 μl of NaCO_3_ solution. Similarly, 20 μl of plant extracts were mixed with the same reagent and the absorbance was measured. Total phenolic contents were expressed as gallic acid equivalents (mg GAE/g sample). Total flavonoids were measured using Aluminum Chloride Colorimetric Method and total content of flavonoids was expressed as quercetin equivalents (mg of QE/g sample) [21].

### Antioxidant activity determination

#### DPPH radical scavenging activity

180 μl of DPPH solution was added to a 20 μl of sample solution and activity was measured at different concentrations (10, 20 and 40 μg/ml). After 60 min, absorbance at 515 nm was measured and the percentage of radical scavenging activity was determined [22]:

$$ \%\mathrm{scavenging}\  \mathrm{activity}=\frac{{\mathrm{Abs}}_{\mathrm{control}}-{\mathrm{Abs}}_{\mathrm{sample}}}{{\mathrm{Abs}}_{\mathrm{control}}}\times 100 $$

Abs_control_ is absorbance of the DPPH solution without sample and Abs_sample_ is absorbance of tested plants. Ascorbic acid was used as a standard antioxidant. IC50 value was also measured.

#### ABTS radical scavenging assay

ABTS radical cation was prepared by mixing 7 mM ABTS stock solution with 2.45 mM Potassium persulphate and after 12–16 h, ABTS^+^ solution was diluted to an absorbance of 0.700 ± 0.02 at 734 nm. 160 μl of ABTS^+^ solution and 10 μl of different plant concentrations were mixed and absorbance was taken at 734 nm and IC50 values were determined [23].

#### NBT (superoxide radical scavenging) assay

1 mL Na_2_CO_3_ and 0.4 mL NBT were added in 1 mL of each extract solution. Then 0.2 mL EDTA solution and 0.4 mL mM hydroxylamine hydrochloride were added. After 15 min of incubation at 25 °C, absorbance was taken at 540 nm. % inhibition and IC50 value will be determined.

#### Reducing power assay

200 μL extract was added with 500 μL phosphate buffer and 500 μL potassium ferricyanide. Reaction mixture was incubated at 50^∘^C and TCA (500 μL) was added and centrifuged for 10 min. Supernatant was mixed with 100 μL of 0.1% FeCl_3_ and the absorbance was measured [24].

#### Cupric ions reducing antioxidant capacity (CUPRAC)

10 μl of 0.01 M CuCl_2_ solution, 10 μl of 7.5 mM ethanol neocuproine solution, and 10 μl of 1.0 M ammonium acetate buffer solution were added to 20 μl of gallic acid and plant extracts. Finally, total volume was adjusted to 1 mL with dH_2_O and absorbance was measured at 515 nm [25].

#### Phosphomolybdate assay

TAC was evaluated by method proposed by Prieto et al. [26]. In this assay, 50 μl of extract was incubated with 500 μl of reaction mixture (28 mM sodium phosphate, 0.6 M sulfuric acid and 4 mM ammonium molybdate) for 90 min at 95 °C in water bath, and absorbance was measured.

### Antioxidant activity index (AAI)

Antioxidant activity index was calculated as it denotes the average of the results based on the six methods used for evaluation of antioxidant activity, namely, DPPH, ABTS, SOR, TRP, CUPRAC and phosphomolybdate assay [27].

### DNA damage protection assay

3 μL of p1391Z plasmid DNA was mixed with 5 μL of Fenton’s reagent and different concentrations of plant extracts and the final volume was brought up to 15 μL using dH_2_O. The reaction mixtures were then incubated for 30 min at 37 °C and bromophenol blue dye was added. The reaction mixtures (15 μL) were loaded on 0.4% agarose gel and electrophoresis was carried out followed by ethidium bromide staining. After that DNA bands were analyzed [28].

### Antimicrobial assay

Bacterial and fungal strains were collected from the Department of Microbiology, Quaid-i-Azam University, Islamabad and propagated in Plant Biochemistry and Molecular Biology Laboratory, Quaid-i-Azam University, Islamabad, Pakistan. Bacterial strains include *S. aureus* (ATCC 2593), *L. monocytogenes* (ATCC 13932), *B. spizizenii* (ATCC 6633), *S. typhi* (ATCC 1428) and *E. coli* (ATCC 25922). The fungal pathogens include *W. anomalus*, *F. oxysporum*, *Mucor* sp., *A. flavus*, *A. niger* and *S. cerevisae.* Bacterial isolates were sub-cultured in a nutrient broth and then incubated at 37 °C for 18 h while the fungal isolates were sub-cultured on SDA media for 24 h at 25 °C. Antibacterial and antifungal assay were performed via agar disc diffusion method. Oxytetracycline and Chloramphenicol were used as a positive control [29, 30]. The zones < 8 mm were not considered significant. MIC and Activity index for each extract was calculated.

### Cytotoxic brine shrimp assay

Six different concentrations of plant extracts (i.e 6, 12, 25, 50, 100 and 250 μg/mL) were added in each vial and their final volume was brought to 5 mL with the help of saline solution. After 24 h, ten shrimps were transferred to each vial and were incubated at 32 **°**C for 24 h, after which the survivors were counted [31]. LC50 values and % mortality were calculated.

### Statistics

Each experiment was performed at least three times, and mean ± standard error (SE) was measured. Results of phytochemical tests and antioxidant activity were subjected to ANOVA using Statistix version 8.1 and comparison among mean values was made by LSD [32]. Graphpad prism was used to calculate IC50 values. LC50 values were calculated using probit analysis program and were assessed at 95% confidence interval [33]. Densitometry calculation of DNA bands was done with the help of ImageJ software.

## Results

### Qualitative analysis of phytochemicals and determination of total phenols and flavonoids contents

Preliminary phytochemical tests of selected plants revealed that various secondary metabolites are strongly present in most of the ethanol plant extracts as compared to the acetone plant extracts. Flavonoids and phenols were strongly present in all selected plant extracts while alkaloids and terpenoids were weakly present in all plant extracts. Glycosides and steroids were present in all species except in DiAAE and ElIAE. Anthocyanin and saponins were detected in all samples except in SaSAE. Tannins was strongly present in DaAEE, DaAAE, ImCEE and ImCAE as compared to the other plants excluding PoAEE and PoAAE in which tannins were not present. However, coumarins were found in all plant extracts except in SaSAE (Table 1).

**Table 1:** Qualitative analysis of phytochemicals of selected plants

Phytochemi-cals	DiAEE	DiAAE	ElIEE	ElIAE	PoAEE	PoAAE	DaAEE	DaAAE	SaSEE	SaSAE	VeZEE	VeZAE	ImCEE	ImCAE
Alkaloids	++	+	+++	++	+	–	+++	+++	–	–	+++	+	+++	+
Flavonoids	+++	++	+++	++	++	++	+++	+++	+++	+++	+++	+++	+++	++
Glycosides	+++	–	+++	–	++	++	++	++	+++	++	++	++	++	++
Phenols	++	+	+++	++	++	+	+++	+++	+++	+++	+++	++	+++	+
Steroids	++	–	+	–	++	+	+	++	+++	++	+++	++	++	++
Terpenoids	+	+	+	+	+	+	++	+	++	+	++	+	++	+
Saponins	+++	–	+++	–	+++	–	+++	+	++	+	+++	++	+++	+
Tannins	+	+	+	++	–	–	+++	+++	++	+	+	+	+++	++
Anthocyanin	+++	+++	+++	+++	+++	+	+++	+++	+	–	++	+	++	+
Coumarin	++	+	+++	++	+++	+	+++	+	+++	–	+++	++	+++	++

+++ Strongly present; ++Moderately present; + Weakly present; − Absent

**Fig. 1:**
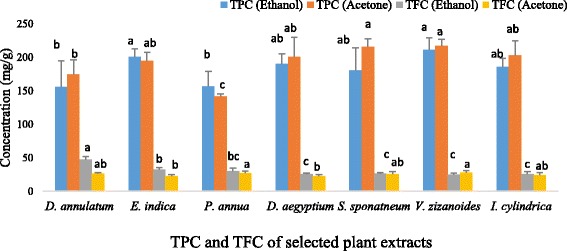
TPC and TFC in selected plant extracts. Data represents the mean of three replicates and each letter (a-c) indicates significance at *P* < 0.05

The quantitative analysis of the selected plant extracts revealed that in case of ethanol plant extracts VeZEE and ElIEE (211.017 ± 18.51 mg GAE/g and 201.115 ± 11.61 mg GAE/g) exhibited maximum phenolic contents while DiAEE and ElIEE (47.023 mg/g ± 4.56 mg QE/g and 32.621 ± 2.89 mg QE/g) revealed maximum flavonoids contents. However, among acetone plant extracts, VeZAE and SaSAE (217.022 ± 10.38 mg GAE/g and 215.723 ± 11.98 mg GAE/g) possess maximum phenolic contents while VeZAE and PoAAE (28.108 ± 2.55 mg QE/g and 27.385 ± 2.88 mg QE/g) exhibited maximum flavonoid contents (Fig. 1).

### Antioxidant activities

Antioxidant activity is a parameter used to assess therapeutic efficacy of plants as it scavenges free radicals in a specific reaction medium. The free radicals including DPPH, ABTS and SOR scavenging activity of respective plant extracts was determined and compared with standard antioxidant (ascorbic acid). In DPPH assay, IC50 value of ascorbic acid (positive control) was recorded as 16.913 ± 2.57 μg/ml. IC50 values of the selected species for radicals scavenging activities are presented in Table 2. The lower the IC50, the stronger the scavenging activity. In present studies, among ethanol plant extracts DiAEE showed stronger DPPH radical scavenging activity (IC50 32.803 ± 9.66 μg/ml) while PoAEE showed minimum DPPH scavenging activity (IC50 107.506 ± 9.60 μg/ml). In case of acetone plant extracts, VeZAE showed stronger DPPH radical scavenging activity (IC50 78.48 ± 7.79 μg/ml) while ImCAE showed minimum DPPH scavenging activity (IC50 408.9 ± 8.16 μg/ml). Moreover, analysis of variance indicated significant variations among various plant species.

**Table 2:** Evaluation of IC50 values of free radical scavenging activities

Plant material	DPPH radical scavenging activity		ABTS radical scavenging activity		SOR scavenging activity	
	Ethanol	Acetone	Ethanol	Acetone	Ethanol	Acetone
*D. annulatum*	32.803 ± 9.66^D^	390.4 ± 5.40^B^	3.897 ± 1.21^D^	31.26 ± 8.73^B^	64.526 ± 9.41^B^	70.403 ± 3.98^D^
*E. indica*	92.626 ± 8.13^B^	294.766 ± 2.70^C^	11.365 ± 3.70^B^	26.82 ± 5.58^BCD^	70.606 ± 9.28^B^	44.013 ± 5.45^F^
*P. annua*	107.506 ± 9.60^A^	102.016 ± 8.77^E^	8.334 ± 0.03^BC^	50.793 ± 9.74^A^	68.41 ± 8.97^B^	237.8 ± 8.88^A^
*D. aegyptium*	85.466 ± 5.97^B^	227.133 ± 4.79^D^	22.716 ± 3.14^A^	17.093 ± 2.43^D^	70.296 ± 9.43^B^	49.27 ± 5.60^EF^
*S. spontaneum*	53.21 ± 5.22^C^	384.2 ± 9.65^B^	3.696 ± 3.30^D^	19.963 ± 4.13^CD^	71.57 ± 8.86^B^	58.063 ± 6.90^E^
*V. zizanioides*	45.53 ± 7.75^CD^	78.48 ± 7.79^F^	10.988 ± 2.62^B^	34.206 ± 7.44^B^	105.833 ± 5.15^A^	98.706 ± 8.87^B^
*I. cylindrica*	35.686 ± 7.26^D^	408.9 ± 8.16^A^	4.267 ± 2.45^CD^	28.31 ± 6.85^BC^	70.96 ± 8.04^B^	86.606 ± 7.96^C^
Ascorbic acid	16.913 ± 2.57^E^	16.913 ± 2.57^G^	2.804 ± 0.29^D^	2.804 ± 0.29^E^	32.25 ± 4.67^C^	32.25 ± 4.67^G^

Each value in the table is represented as mean ± SD (*n* = 3). Values in the same column followed by a different letter (a-g) are significantly different (*p* < 0.05). (DPPH = 2,2-diphenyl-1picrylhydrazyl; ABTS = 2, 2′-azino-bis (3- ethylbenzthiazoline-6-sulphonic acid); SOR Superoxide radical)

IC50 values of positive control (ascorbic acid) in ABTS assay and SOR assay was recorded as 2.804 ± 0.29 μg/ml and 32.25 ± 4.67 μg/ml. In ABTS radical scavenging activity, maximum activity was detected in ethanol extract of SaSEE (IC50 3.696 ± 3.30 μg/ml) and minimum activity was observed in DaAEE (IC50 22.716 ± 3.14 μg/ml). However, among acetone plant extracts, maximum radical scavenging activity was revealed in DaAAE (IC50 17.093 ± 2.43 μg/ml) and minimum activity was found in PoAAE (IC50 50.793 ± 9.74 μg/ml). In SOR assay, among ethanol plant extracts, maximum scavenging activity was present in DiAEE (IC50 64.526 ± 9.41 μg/ml) and minimum activity was observed in VeZEE (IC50 105.833 ± 5.15 μg/ml). However, in case of acetone extracts, maximum activity was detected in ElIAE (IC50 44.013 ± 5.45 μg/ml) and minimum activity in PoAAE (IC50 237.8 ± 8.88 μg/ml). Overall, ethanol plant extracts showed remarkably stronger free radicals scavenging activity as compared to the acetone plant extracts. Moreover, strong SOR scavenging activity of DiAEE, ElIEE and ElIAE statistically similar to the standard ascorbic acid revealed strong antioxidant fractions in these extracts (Table 2).

**Fig. 2:**
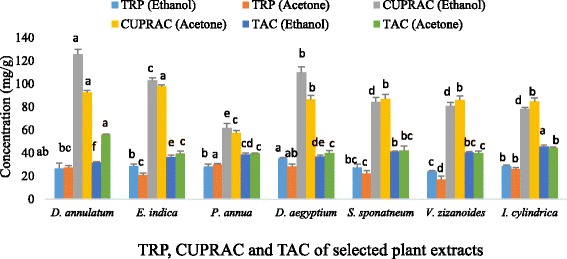
TRP, CUPRAC and TAC of the selected plant extracts. Data represents the mean of three replicates and each letter (a-f) indicates significance at *P* < 0.05 (Total reducing power and total antioxidant capacity expressed as ascorbic acid equivalent (mg AE/g extract); Cupric ions reducing assay expressed as gallic acid equivalent (mg GA/g extract))

In reducing power assay and CUPRAC assay, the higher absorbance indicates stronger antioxidant activity. Among ethanol plant extracts, maximum reducing power ability was found in DaAEE (35.107 ± 1.07 mg/g) and minimum in VeZEE (23.889 ± 0.87 mg/g). However, among acetone plant extracts, maximum reducing power ability was found in PoAAE (30.029 ± 0.77 mg/g) and minimum in VeZAE (17.009 ± 2.86 mg/g) respectively (Fig. 2). Similarly, cupric ion reducing power of the ethanol plant extracts ranked in decreasing order of DiAEE > DaAEE > ElIEE > SaSEE > VeZEE > ImCEE > PoAEE (125.281 ± 4.53, 109.718 ± 4.75, 102.953 ± 1.9, 84.129 ± 3.96, 80.796 ± 2.8, 78.174 ± 1.34, 61.850 ± 3.78 mg/g). However, cupric ion reducing power of the acetone plant extracts was found in decreasing order of ElIAE > DiAAE > SaSAE > DaAAE > VeZAE > ImCAE > PoAAE (97.438 ± 1.38, 92.365 ± 1.84, 86.580 ± 4.19, 86.482 ± 3.42, 85.723 ± 3.96, 84.914 ± 2.73, 57.389 ± 1.97 mg/g) respectively (Fig. 2). These values of CUPRAC assay were determined from the regression equation obtained from positive control i.e. gallic acid (y = 0.0136× + 0.0845).

The results obtained from phosphomolybdate assay show that ethanol extract has potent ability to reduce Mo(VI) to Mo(V) as compared to the acetone extracts. In comparing ethanol plant extracts, total antioxidant capacity was observed maximum in ImCEE (45.815 ± 0.86 mg/g) and minimum in DiAEE (31.626 ± 0.72 mg/g) while among acetone plant extracts total antioxidant capacity was found maximum in DiAAE (55.807 ± 0.62 mg/g) and minimum in ElIAE (39.502 ± 2.26 mg/g). These values were determined from the regression equation obtained from standard ascorbic acid curve (y = 0.0405× + 0.3588). Analysis of variance also showed significant variations among selected plant species (Fig. 2).

### Antioxidant activity index (AAI)

The Antioxidant Activity Index was used to rate the selected plant species on the basis of antioxidant potential. Some plants showed variable antioxidant potential in different assays. For example, DiAEE was revealed as a potent antioxidant by DPPH scavenging assay, ABTS scavenging assay, SOR scavenging assay and Cupric ions reducing assay but moderate or weak antioxidant by total antioxidant assay and total reducing power assay. Owing to such difficulty in analyzing results from each assay, Antioxidant Activity Index (AAI) in terms of percentage was determined to combine the average results of six assays for evaluation of antioxidant capacity.

**Fig. 3:**
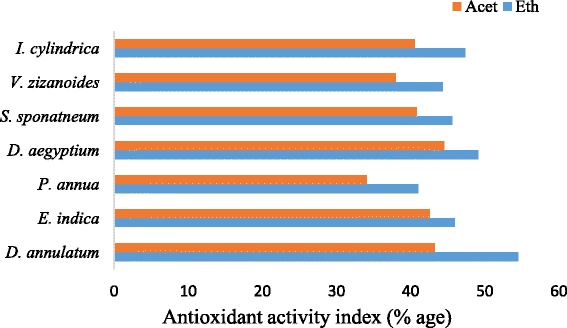
Antioxidant activity index (AAI) of ethanol and acetone extracts of selected plant species

Present studies revealed that among both ethanol and acetone plant extracts, DiAEE, DiAAE, DaAEE and DaAAE (AAI- 54.54%, 43.24%, 49.13% and 44.52%) showed highest relative antioxidant potential while PoAEE and PoAAE (AAI- 41.04% and 34.11%) showed poor antioxidant activity. Highest antioxidant activity in *D. annulatum* and *D. aegyptium* was significantly correlated with phytochemicals as they possess maximum phenols and flavonoids contents while *P. annua* possess minimum antioxidant potential due to presence of less phenols and flavonoids contents. However, other plant species showed relatively moderate antioxidant potential. Overall, ethanol plant extracts exhibit maximum antioxidant activity as compared to the acetone plant extracts as shown in Fig. 3. It might be due to the chemical compounds present in these species.

### DNA damage protection assay

**Fig. 4:**
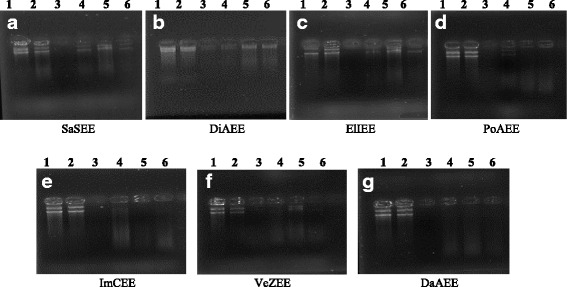
Effects of the different ethanol plant extracts against oxidative damage to DNA (p1391Z). (Lane 1: Control genomic DNA; Lane 2: Fenton’s reagent + DNA; Lane 3: Fenton’s reagent + DNA + Quercetin; Lane 4: Fenton’s reagent + DNA + 2 μl plant extract; Lane 5: Fenton’s reagent + DNA + 4 μl plant extract; Lane 6: Fenton’s reagent + DNA + 6 μl plant extract)

To access the prevention of oxidative DNA damage by the plant extracts, p1391Z supercoiled DNA was used. Control untreated DNA showed two bands, one of open circular DNA and one of supercoiled DNA. Combine treatment of DNA with Fenton reagent and quercetin (positive control) maintained the supercoiled DNA from scission while treatment of DNA with Fenton reagent in the absence of plant extracts led to the strand scission of the supercoiled DNA. DNA treated with different concentrations of plant extracts showed very little DNA damage protection activity (Fig. 4).

**Fig. 5:**
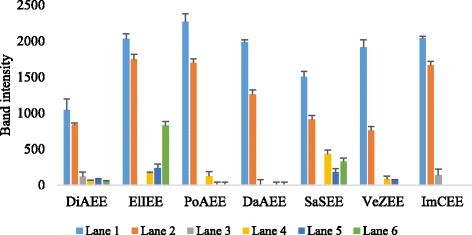
Band intensity graph of DNA protecting activity. (Lane 1: Control genomic DNA; Lane 2: Fenton’s reagent + DNA; Lane 3: Fenton’s reagent + DNA + Quercetin; Lane 4: Fenton’s reagent + DNA + 2 μl plant extract; Lane 5: Fenton’s reagent + DNA + 4 μl plant extract; Lane 6: Fenton’s reagent + DNA + 6 μl plant extract)

Maximum DNA damage prevention was shown by SaSEE and DiAEE followed by ElIEE while other plants did not reveal any DNA damage preventive activity. It was also noted that ethanol extracts possess greater flavonoids and phenols contents so they revealed some DNA damage protection activity while acetone extracts exhibit less phenols and flavonoids contents so they were not found effective for DNA damage protection assay. The DNA damage prevention was increased with increase in plant concentration. Densitometric analysis confirmed the experimental data (Fig. 5).

### Antimicrobial assay

Antimicrobial activity of the plant extracts was observed against nine tested microorganisms. Oxytetracycline was used as a standard drug against bacterial strains and Chloramphenicol was used as a standard drug for fungal strains. No activity was observed against two microorganisms (*F. oxysporum* and *A. niger*) in both ethanol and acetone plant extracts. Most susceptible organism in the studies was *A. flavus* followed by *B. spizizenii, Mucor* specie and *W. anomalous*. The range of minimum inhibitory concentration (MIC) of extracts recorded was 25–75 μg/ml (Table 3).

**Table 3:** Antimicrobial activity determined as zone of inhibition (mm ± SD) and minimum inhibitory concentration (μg/ml) of selected plant extracts against selected microbial strains

Plant extracts		DiAEE	DiAAE	ElIEE	ElIAE	PoAEE	PoAAE	DaAEE	DaAAE	SaSEE	SaSAE	VeZEE	VeZAE	ImCEE	ImCAE	Control
*E. coli*	ZOI	NI	NI	7 ± 0.5	NI	NI	NI	7 ± 0.5	5 ± 4.1	NI	4 ± 3.4	4 ± 3.4	NI	7 ± 0.5	5 ± 4.1	28 ± 2.0
	MIC	–	–	–	–	–	–	–	–	–	–	–	–	–	–	–
*S. aureus*	ZOI	8 ± 0.5	NI	8 ± 0.5	NI	NI	8 ± 0.5	8 ± 1.1	NI	NI	NI	NI	NI	NI	NI	33 ± 1.1
	MIC	75	–	75	–	–	75	75	–	–	–	–	–	–	–	–
*L. monocyto-genes*	ZOI	7 ± 0.5	8 ± 1.0	8 ± 1.1	8 ± 0.5	NI	NI	7 ± 1.1	8 ± 1.0	NI	5 ± 4.0	8 ± 1.5	5 ± 4.3	8 ± 2.3	8 ± 0.5	31 ± 1.5
	MIC	–	75	75	75	–	–	–	75	–	–	75	–	75	75	–
*B. spizizenii*	ZOI	8 ± 0.5	NI	9 ± 2.8	4 ± 3.4	8 ± 0.5	8 ± 2.6	8 ± 1.5	8 ± 1.1	6 ± 0.5	5 ± 5.0	8 ± 2.3	8 ± 0.5	8 ± 1.1	7 ± 1.1	28 ± 4.0
	MIC	75	–	50	–	75	75	75	75	–	–	75	75	75	–	–
*S. typhi*	ZOI	4 ± 3.7	5 ± 4.0	7 ± 1.1	4 ± 3.7	NI	NI	4 ± 3.78	7 ± 1.0	NI	NI	NI	NI	NI	6 ± 0.5	27 ± 2.3
	MIC	–	–	–	–	–	–	–	–	–	–	–	–	–	–	–
*W. anomalus*	ZOI	7 ± 0.5	7 ± 1.0	7 ± 1.0	8 ± 2.0	NI	7 ± 1.0	7 ± 1.5	7 ± 1.0	7 ± 1.0	7 ± 1.5	7 ± 1.5	7 ± 1.0	7 ± 1.0	7 ± 1.0	30 ± 2.6
	MIC	–	–	–	75	–	–	–	–	–	–	–	–	–	–	–
*F.oxysporum*	ZOI	NI	NI	NI	NI	NI	NI	NI	NI	NI	NI	NI	NI	NI	NI	32 ± 1.8
	MIC	–	–	–	–	–	–	–	–	–	–	–	–	–	–	–
*Mucor*	ZOI	8 ± 1.1	4 ± 3.7	8 ± 1.0	7 ± 1.1	8 ± 1.5	NI	10 ± 2.0	7 ± 1.0	9 ± 2.5	5 ± 4.1	4 ± 3.7	7 ± 1.5	9 ± 3.0	9 ± 1.5	31 ± 1.0
	MIC	75	–	–	–	–	–	50	–	75	–	–	–	75	75	–
*A. flavus*	ZOI	8 ± 1.1	7 ± 1.1	7 ± 0.5	4 ± 3.4	4 ± 3.4	7 ± 1.1	8 ± 1.52	8 ± 2.0	9 ± 2.5	5 ± 4.1	8 ± 1.5	7 ± 1.5	10 ± 1.7	5 ± 4.0	28 ± 2.0
	MIC	75	–	–	–	–	–	75	75	75	–	75	–	50	–	–
*A. niger*	ZOI	NI	NI	NI	NI	NI	NI	NI	NI	NI	NI	NI	NI	NI	NI	33 ± 1.8
	MIC	–	–	–	–	–	–	–	–	–	–	–	–	–	–	–
*S. cerevisiae*	ZOI	11 ± 3.0	7 ± 1.0	NI	NI	NI	NI	12 ± 5.1	11 ± 3.7	9 ± 1.7	9 ± 1.5	11 ± 3.5	11 ± 4.9	12 ± 4.0	11 ± 4.9	30 ± 2.0
	MIC	–	–	–	–	–	–	50	50	75	75	50	50	50	50	–

*ZOI* Zone of inhibition (mm ± SD), *MIC* Minimum inhibitory concentration (μg/ml), *NI* No inhibition; Positive control: Oxytetracycline (Bacterial strains) and Chloramphenicol (Fungal strains)

In antibacterial activity, majority of the plant extracts showed little or non-significant activity against *S. aureus, S. typhi* and *E. coli*. Among ethanol plant extracts, ElIEE showed maximum activity (ZOI: 9 mm, AI: 0.321) against *B. spizizenii* while among acetone plant extracts most of the plant species showed maximum antibacterial activity (ZOI: 8 mm, AI: 0.258) against some bacterial strains. Among bacterial strains, lowest MIC values (50 μg/ml) were recorded for ElIEE (against *B. spizizenii*). Similarly, in antifungal activity DaAEE (ZOI: 12 mm, AI: 0.4) as well as DaAAE (ZOI: 11 mm, AI: 0.366) showed maximum activity against *S. cerevisae* as compared to the other plant extracts. However, no inhibition zones were observed against *F. oxysporum* and *A. niger*.

**Fig. 6:**
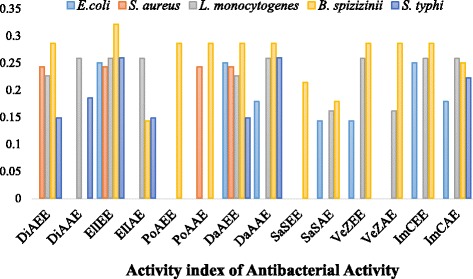
Activity Index of antibacterial activity of selected ethanol and acetone plant extracts

**Fig. 7:**
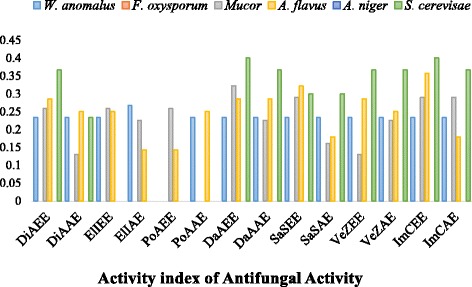
Activity Index of antifungal activity of selected ethanol and acetone plant extracts

Overall, maximum antimicrobial activities were recorded for DaAEE (i.e against nine tested microbial strains) and DaAAE and ImCAE (i.e against eight microbial strains) and minimum activity was recorded for PoAEE (i.e against three strains only) and PoAAE (i.e against four strains only) respectively. Significant correlation was observed between antimicrobial activity and total phenols and flavonoids contents. As PoAEE and PoAAE possess minimum phenols and flavonoids contents, so they revealed less antimicrobial efficacy. However, remaining plant extracts revealed some activity against few strains as shown in Figs. 6 , 7.

### Cytotoxic activity

The ethanol and acetone extracts of the selected plants revealed good brine shrimp cytotoxic activity. Lethal concentration (LC50) in ethanol plant extracts ranged from 11.004 to 20.083 ppm and in case of acetone plant extracts ranged from 7.932 to 23.090 ppm respectively. The degree of lethality was directly related to the concentration of extract. Maximum mortalities (100%) were observed at a concentration of 250 ppm in both ethanol and acetone extracts. The observed lethality of the plant extracts to brine shrimps revealed the presence of cytotoxic compounds in these plants.

Vincristine sulfate was used as a positive control and its LC50 value was recorded as 0.839 ppm. Results revealed that among ethanol plant extracts, ImCEE, DiAEE and SaSEE (LC50 11.004 ppm, 11.223 ppm and 11.488 ppm) showed remarkably significant cytotoxic potential and PoAEE (LC50 20.083 ppm) showed minimum cytotoxicity. Whereas remaining ethanol extracts revealed moderate cytotoxic activity. In case of acetone plant extracts, good cytotoxic potential was observed in ImCAE and VeZAE (LC50 7.932 ppm and 8.877 ppm) and least cytotoxic potential was observed in SaSAE (LC50 23.090). However, other species showed moderate cytotoxic potential (Table 4).

**Table 4:** Percentage mortality of brine shrimps in probits at five different concentrations and respective LC50 values

Plant extracts	Mortality (%) in Probits at different doses					Slope	Intercept	R square	LC50	95% CI
	6	12	25	50	100					
DiAEE	4.75	5.00	5.41	5.52	5.95	0.958	3.994	0.943	11.223	4.905–25.680
DiAAE	4.75	4.82	5.25	5.33	6.28	1.128	3.707	0.905	14.027	7.013–28.058
ElIEE	4.48	4.75	5.08	5.52	6.48	1.518	3.137	0.951	16.857	9.783–29.043
ElIAE	4.90	5.15	5.25	5.71	6.75	1.319	3.691	0.910	9.828	5.256–18.378
PoAEE	4.48	4.56	5.08	5.25	6.28	1.358	3.231	0.925	20.083	11.206–35.992
PoAAE	4.48	4.75	5.25	5.33	6.28	1.341	3.343	0.940	17.220	9.444–31.398
DaAEE	4.26	4.90	5.08	5.25	6.28	1.398	3.197	0.931	19.455	10.940–34.597
DaAAE	4.64	4.90	5.25	5.41	6.75	1.439	3.354	0.900	13.936	7.977–24.346
SaSEE	4.75	5.15	5.25	5.33	6.28	1.020	3.918	0.886	11.488	5.361–24.618
SaSAE	3.87	4.75	5.00	5.52	–	1.673	2.719	0.969	23.090	13.974–38.155
VeZEE	4.64	4.90	5.15	5.61	6.75	1.532	3.256	0.929	13.749	8.002–23.624
VeZAE	4.90	5.00	5.52	5.84	–	1.089	3.967	0.956	8.877	4.153–18.972
ImCEE	4.75	5.15	5.25	5.61	6.48	1.245	3.704	0.933	11.004	5.698–21.251
ImCAE	5.00	5.25	5.41	5.84	6.75	1.286	3.844	0.929	7.932	4.095–15.366

*LC50* Lethal concentration fifty, *CI* Confidence interval

## Discussion

Herbal medicines are considered as one of the most important fields of traditional medicine all over the world. Treatment using natural medicines is gaining attention nowadays because of harmful synthetic additives used by individuals. Sometimes, stem of *S. spontaneum* is chewed to relieve stomach pain and the whole plant of *D. annulatum* is used for dysentery and manorrhagia [7, 34]. Similarly, the ethnomedicinal importance of *E. indica, D. aegyptium, V. zizanoides* and *I. cylindrica* has been well reported [35–39] but the biological activities of majority of these species have not been investigated. Present studies revealed that majority of these species contain various secondary metabolites in varying concentrations (Table 1) as reported by Ratha et al. [39], Abbas et al. [40], Babu and Savithramma [41], Devi and Kottai [42] and Alaekwe [43]. Quantitative analysis revealed that the phenolic contents were found in greater concentration in all plants as compared to the flavonoids contents (Fig. 1). Phenol and flavonoid compounds exhibit inhibitory effects against multiple viruses and bacteria and possess free radical scavenging and anticancer activity. Similarly, saponins have potent antimicrobial, antioxidant and cytotoxic potential in plants [44, 45]. Hundreds of steroids are also present in plants which play crucial role in many disorders such as prostrate cancer. Anthocyanins belong to parent class of molecules called flavonoids and possess significant scavenging activity in plants. Many plants also store chemicals in the form of cardiac glycosides which are used in the treatment of heart diseases while alkaloids have strong antimicrobial efficacy [46, 47]. Coumarins and terpenoids are active against viruses, bacteria and fungi [48, 49]. Hence, presence of these secondary metabolites in plants indicates significant therapeutic potential in plants.

Natural antioxidants protect us from various diseases by scavenging ROS [50]. Antioxidants in crude plant extracts have multifunctional activities, thus a single antioxidant activity assay might be inadequate to predict and measure the antioxidant efficacy of natural antioxidants. The utilization of assays that measure free radical scavenging activity in combination with reducing power assays is recommended for the determination of natural antioxidant potential. Therefore, six different antioxidant assays, i.e., DPPH, ABTS, SOR, TRP, CUPRAC and phosphomolybdate assays, were conducted to determine the antioxidant efficacy of some commonly occurring grass species. Results revealed that DiAEE, DiAAE, DaAEE and DaAAE (AAI- 54.54%, 43.24%, 49.13% and 44.52%) showed maximum antioxidant activity while PoAEE and PoAAE (AAI- 41.04% and 34.11%) showed least antioxidant activity. However, moderate antioxidant activity was revealed in other plants (Fig. 3). Positive results in all the antioxidant activity assays used in this study indicate that the various compounds in the crude extracts could act as free radicals’ scavenger viz., DPPH, ABTS, SOR or possess the reducing power potential of Fe^3+^, Cu^2+^ and Mo (IV) complex. Devi and Kottai [42], Iqbal and Gnanaraj [51] and Rekha and Shivanna [52] also reported antioxidant activity in *S. spontaneum, E. indica* and *D. aegyptium.* Intake of such antioxidants protect the body from damage caused by harmful molecules such as free radicals, ultimately lowering the risk of infections and some forms of cancer and so, improves the health.

Hydroxyl radicals may also cause DNA strand breakage, which may further cause mutagenesis, carcinogenesis and cytotoxicity [47]. DNA protective activity of selected plant extracts was assessed on DNA strand breakage using genomic DNA. Different concentration of plant extracts was treated with fenton’s reagent and DNA bands were analyzed. Band intensity was also measured to check the effectiveness of plants (Fig. 5). The results proved that SaSEE, DiAEE and ElIEE protects DNA from damage while other species didn’t exhibit DNA protecting activity. Present results are in accord with those of Sagnia et al. [53] who confirmed that ElIEE possess significant superoxide radical scavenging activity and has ability to protect DNA damage. Keeping in mind the toxic effects produced by different solvents used for extraction, ethanol was carefully chosen to check the DNA protection ability of plants as the ethanol extraction product mainly contains high hydrophilic compounds and hence possess less toxic effects to living cells.

Antimicrobial is an agent that destroy or inhibits the growth of microbes such as bacteria, fungi, or viruses, as well as protozoans. Hence, now-a-days antimicrobials of plant origin are generally recommended as they are more effective in treatment of infectious diseases simultaneously alleviating many side effects which are associated with synthetic antibiotics [54]. The antimicrobial activity was performed by agar disc diffusion method. Results revealed that most of the plant extracts were more active against *L. monocytogenes, W. anomalous, A. flavus* and *Mucor* specie while *S. aureus, F. oxysporum* and *A. niger* were revealed as highly resistant pathogens. Overall, all plant extracts showed moderate to weak activity against most of the tested pathogens (Table 3). These results are supported by the findings of Parkavi et al. [55] and Kumar et al. [10] who revealed significant antibacterial activity of ImCEE and DaAEE against *E. coli*. Similarly, Fazal et al. [56] also reported the antimicrobial efficacy of VeZEE. Moreover, Morah and Otuk [57] documented significant activity of the ElIEE against *E. coli, S. typhi* and *S. aureus*. Crude extracts of these plants can be used as an alternative drug to control food poisoning and other infectious diseases caused by resistant microbes and to preserve foodstuff as well.

Cytotoxic drugs are highly toxic to cells, especially through their action on cell reproduction. These drugs are increasingly being used for the treatment of cancer, multiple sclerosis, autoimmune disorders and many other diseases. Cytotoxic materials are generally identified by a purple symbol that shows a cell in late telophase [58]. It was interest of this study to determine cytotoxicity activity of these species using brine shrimp lethality test as it is considered as the inexpensive, rapid and simple bioassay for cytotoxicity study of the plant extracts. Previous literature [59] provides strong evidence that plant extracts with LC50 values below 20 μg/ml have more probability of producing anticancer compounds. In present studies, all plant extracts revealed strong to moderate cytotoxic potential (Table 4). These results are supported by the findings of Wong et al. [60] and Responte al et. [61] who demonstrated brine shrimps’ cytotoxic potential in *I. cylindrica* and *E. indica*. The cytotoxicity exhibited by the crude extracts indicates the presence of active or potent bioactive compounds in plants which reveals that they possess significant antifungal effects, antibacterial effects and other pesticidal effects.

## Conclusion

Reactive oxygen species (ROS) play an important role in oxidative DNA damage which may causes dysfunction in biological processes in the human body leading to many chronic diseases. Use of herbal medicines to treat different diseases is also preferred as these are cheap, safe and without any harmful side-effects as compared to the synthetic drugs. Similarly, the replacement of synthetic drugs with natural antioxidants may be valuable. To date, biological potential of many grasses of Bahawalpur region have not been evaluated. Hence, in present studies, biological potential of aerial parts of some grasses was checked and comparison among ethanol (polar solvent) and acetone (semi-polar solvent) extracts was made. Results revealed that aerial parts of *D. annulatum, D. aegyptium*, *I. cylindrica, E. indica, S. spontaneum* and *V. zizanioides* possess good antioxidant and antimicrobial activity while *P. annua* did not show significant biological potential. Maximum activities were observed in their ethanol plant extracts as compared to the acetone plant extracts as ethanol extract has greater ability to dissolve polar substances. This study provides experimental evidence that the extracts of these species can be used in crude form to combat various bacterial and fungal infections and can be used as natural antioxidants as well. So, these species possess vital medicinal value rather than being utilized by local individuals and as forage for horses and camels. Selected species contain various secondary metabolites which can be the major contributor to their antibacterial, antifungal, antioxidant and brine shrimps’ cytotoxic activities. These compounds can also be isolated from crude extracts for use in medicinal drugs. However, they are revealed as less effective for DNA damage protection assay. In future, there is a need of establishing a more systemic study to validate the human consumption of selected species for medicinal use through additional toxicity and isolation and characterization of principle active compounds via HPLC and NMR spectroscopy techniques.

## Abbreviations

AAI: Antioxidant activity index
ABTS: 2,2′-azino-bis (3-ethylbenzothiazoline-6-sulphonic acid)
ANOVA: Analysis of variance
ATCC: American type culture collection
BC: Before christ
CI: Confidence interval
CNS: Central nervous system
Cu^2+^: Copper
CuCl_2_: Cupric chloride
CUPRAC: Cupric reducing antioxidant capacity
DaAAE: *Dactyloctenium aegyptium* acetone extract
DaAEE: *Dactyloctenium aegyptium* ethanol extract
dH_2_O: Distilled water
DiAAE: *Dicanthium annulatum* acetone extract
DiAEE: *Dicanthium annulatum* ethanol extract
DNA: Deoxyribonucleic acid
DPPH: 2,2-diphenyl-1-picrylhydrazyl
EDTA: Ethylenediaminetetraacetic acid
ElIAE: *Eleusine indica* acetone extract
ElIEE: *Eleusine indica* ethanol extract
ELISA: Enzyme-linked immunosorbent assay
Fe^3+^: Iron
FeCl_3_: Ferric chloride
GAE: Gallic acid equivalent
HPLC: High-performance liquid chromatography
IC50: Half maximal inhibitory concentration
ImCAE: *Imperata cylindrica* acetone extract
ImCEE: *Imperata cylindrica* ethanol extract
LC50: Fifty/median lethal concentration
LSD: Least significant difference
MIC: Minimal inhibitory concentration
mM: Millimolar
Mo: Molybdenum
NaCO3: Sodium carbonate
NBT: Nitro-blue tetrazolium
NMR: Nuclear magnetic resonance spectroscopy
PoAAE: *Poa annua* acetone extract
PoAEE: *Poa annua* ethanol extract
Ppm: Parts per million
QE: Quercetin equivalent
ROS: Reactive oxygen species
SaSAE: *Saccharum spontaneum* acetone extract
SaSEE: *Saccharum spontaneum* ethanol extract
SDA: Sabouraud dextrose agar
SOR: Superoxide radical scavenging assay
TAC: Total antioxidant capacity
TCA: Trichloroacetic acid
TFC: Total flavonoid contents
TPC: Total phenol contents
TRP: Total reducing power
VeZAE: *Vetiveria zizanioides* acetone extract
VeZEE: *Vetiveria zizanioides* ethanol extract
ZOI: Zone of inhibition
μl: Microliter
